# IUD self-removal as self-care: Research is needed in low and middle-income countries

**DOI:** 10.3389/fgwh.2022.992639

**Published:** 2022-09-07

**Authors:** Alice F. Cartwright, Amelia C. L. Mackenzie, Rebecca L. Callahan, M. Valeria Bahamondes, Laneta J. Dorflinger

**Affiliations:** ^1^FHI 360, Durham, NC, United States; ^2^Department of Maternal and Child Health, Gillings School of Global Public Health, University of North Carolina at Chapel Hill, Chapel Hill, NC, United States; ^3^FHI 360, Washington, DC, United States

**Keywords:** IUD, LARC (long-acting reversible contraception), self-removal, LMICs (low and middle-income countries), self-care, contraception

## Introduction

The ability to freely decide one's number, spacing, and timing of children has been highlighted as a human right in international declarations and research, policy, and programmatic efforts in family planning (1). Accessing and using one's preferred contraceptive method is also a crucial component of ensuring people's reproductive autonomy or empowerment (2). The publication of the World Health Organization (WHO)'s updated Guidelines on Self-Care for Sexual and Reproductive Health and Rights (SRHR) in 2019 highlighted that self-care interventions for SRHR might be particularly important in supporting people's free, full, and informed-decision making (3). These guidelines underscore that while self-care may facilitate more individual control over one's own reproductive health, these approaches are embedded within larger health systems. Therefore, self-care suggests a balance between supporting people's autonomy as active participants in their own reproductive health decisions, while acknowledging that people may still desire support from healthcare providers. This balance is particularly important for people who may be marginalized, as a result of discriminatory gender norms or unequal power in families or intimate relationships, or gender and sexual minorities. Some members of these groups might welcome self-care to reduce exposure to less-supportive health systems, while others might appreciate more structured care.

## Impact of COVID-19 on contraception access

The timing of the WHO self-care guidelines was particularly prescient as the COVID-19 pandemic caused incredible disruptions to health systems worldwide, including limiting access to health facilities, providers, and services that were not considered “essential.” Threats to reproductive autonomy during this time included recommendations that the provision of long-acting reversible contraceptive (LARC) methods be prioritized over other methods and that removal of LARC be postponed (extended use) to conserve medical resources and reduce unnecessary COVID-19 exposures (4). Preliminary evidence from selected sub-Saharan African (SSA) countries indicated that overall access to family planning services did not appear to be severely disrupted in the early months of the pandemic (5, 6); however, certain populations, including nulliparous women and women living in rural areas, reported a significant increase in need for contraceptive services (defined as those who were sexually active and did not intend to get pregnant in the next 12 months) from the period before to during COVID-19 (5). Initial interruptions to family planning services were followed by subsequent increases in new users in Senegal through December 2020 (7). Future threats to contraceptive care might emerge as the pandemic persists and evolves. Research has not sufficiently documented whether the use of self-care protected against contraceptive stock-outs or interruptions in services, but increasing people's knowledge of and access to self-care approaches seems especially important during the ongoing disruption to health systems.

## Self-care and preferred contraceptive characteristics

Self-care options might also better align with people's contraceptive preferences. Evidence from the United States (US) and many low and middle-income countries (LMICs) reflects that the top user-preferred contraceptive features are effectiveness at preventing pregnancy, ease of use, and causing few or no side effects (8–11). However, another important feature is that the method can be stopped at any time, or that women themselves have control over the discontinuation of the method (8, 12). While control over discontinuation is relatively simple with many short-acting methods (e.g., a person can stop taking oral contraceptive pills or choose not to return to a health facility for a contraceptive injection), discontinuation of LARC requires a clinical visit for removal. As contraceptive implants have soared in popularity across SSA over the past decade, important attention has been raised regarding ensuring the availability of removal services, in line with principles of informed choice and autonomy (13).

## IUD prevalence and acceptability in LMICs

While implants have made up an increasing proportion of the contraceptive method mix over the past decade, especially in SSA and some Latin American countries (14, 15), intrauterine devices (IUDs) continue to be a small fraction of modern contraceptives used in many LMICs (16). To date, copper IUDs have been the predominant type of IUD available globally when compared to hormonal IUDs, and are the most commonly used LARC method in Latin America, Asia, and the Middle East/North Africa (17). However, with the exception of a few countries and specific target groups, they are far less popular in SSA compared with implants (16, 17). And while the use of hormonal IUDs has increased exponentially in the US over the past 20 years (18), the cost of these products has limited their availability in LMICs (19).

IUDs are highly effective at preventing pregnancy over a long duration, a desirable method characteristic (9, 20). However, barriers such as a lack of trained providers, the need for pelvic exams and necessary facility space, lack of instruments and supplies, and low demand have impeded their wider uptake (19). Because the proportion of women using IUDs has historically been so low in SSA, it has been difficult to assess their acceptability. However, research from South Africa has shown the impact of quality, informed counseling, with women randomized to use copper IUDs not any more likely to discontinue the method at 12–18-month follow-up than those using implants or injectable contraception (21). In addition, through partnerships of governments, donors, manufacturers, procurement agencies, and research and service delivery groups, such as the Hormonal IUD Access group (22), pilot introduction programs of hormonal IUDs have shown high acceptability in SSA (23), with 12-month continuation rates over 86% in Nigeria and Zambia and no significant differences in continuation between hormonal- and copper-IUD users (24). The collaborative efforts currently underway to reduce the prices of hormonal IUDs worldwide may foretell a corresponding increase in uptake of the method across LMICs when commodities are more broadly available.

## User preferences and experiences of IUD self-removal and provider opinions

Ahead of the wider introduction, especially of hormonal IUDs in LMICs, it is worth reflecting on lessons learned to date on access to implant removal, but also opportunities to better understand women's preferences regarding autonomy and self-care related to IUD use and discontinuation. Having the option to remove one's own IUD might increase method desirability because women could potentially stop using the method when they want without having to return to a health facility. A US study found increased interest in IUD use when the possibility of self-removal was explained (25), and 54% of IUD users seeking removal were more likely to recommend IUDs to a friend after learning about the option of self-removal (26). The potential for self-removal may be even more attractive to populations who have been disenfranchised or faced stigma at health facilities.

While some alarmist news reports in the US have cited individual examples of IUD self-removals gone wrong (27), growing evidence exists that IUD self-removal *is* happening, and strategies for successfully doing so are being discussed, particularly online. Analyses of online forums in the US and YouTube videos in English show that former IUD users are providing examples of their experiences and tips to others who might want to try self-removal (28–30). These analyses find that many self-removal attempts are motivated by a desire to have control over when to discontinue the method, high costs for removals from some healthcare centers, lack of appointment availability, and provider reluctance/refusal to remove (28).

Formal or clinical research, especially on success rates of IUD self-removal, is limited in the US and, as far as we know, non-existent in LMICs. In one US study, 46% of IUD users were willing and able to feel their IUD strings (a necessary pre-condition for self-removal), either at home or at a clinical visit (31). The only study to date on the success rates of IUD self-removal was conducted at clinics in five US states and found that 59% of IUD users presenting for IUD removal were willing to try self-removal (26). Of those, 19% were able to successfully self-remove their IUDs. Successful self-removal was greater among copper IUD users vs. hormonal IUD users, those who had longer strings, and those who used a squatting or lying down position. In addition, the use of gloves might aid in gripping strings for removal.

Some providers have expressed concern regarding IUD self-removal in qualitative research, news reports, and on social media. One concern is people might remove their own IUDs hastily and then find themselves without a contraceptive method to protect against pregnancy (32). However, a US study found no differences in IUD continuation at 6-months between IUD users who were counseled about self-removal at the time of insertion and those who were not (33). Other complications feared by providers include IUDs embedded in the uterus or breaking of the threads or pieces of the IUD during self-removal (an extremely rare event), though others have countered that people can be provided adequate guidance about what to do if they experience those issues (34). While providers have historically been concerned about inserting IUDs among people who might have active sexually transmitted or other infections due to a small risk of introducing infections into the uterus, it is not clear that *removing* an IUD would have a similar level of infection risk. Finally, some have expressed fears that informing IUD users about self-removal might reduce follow-up visits to provide additional counseling and care. This is in spite of clinical recommendations that routine follow-up visits after IUD insertion are not required (35).

## Discussion

As IUD use continues to increase worldwide, more research on the demand for and interest in IUD self-removal in LMICs is needed. Initial questions include whether the potential for self-removal might increase interest in IUD use in contexts where uptake has historically been low, and if people would still be interested if successful self-removal could not be guaranteed. The acceptability for longer IUD strings needed to facilitate self-removal would also need to be assessed. This is particularly important in the context of discreet IUD use and whether longer strings would be more or less detectable to a sexual partner. In addition, health care providers, program planners, and researchers would benefit from knowing more about when and through what mechanisms IUD users would want to receive self-removal information. Those messages should be informed by rigorous research, rather than hearsay, and include when to stop trying to self-remove, what a partial removal might mean for pregnancy protection, and when to seek care from a health facility. Better understanding provider perspectives on IUD self-removal should include both asking about opinions in large-scale surveys and more in-depth and nuanced research conducted through qualitative methods. For many providers, IUD provision may be new or uncommon, and therefore the idea of IUD self-removal surprising or potentially alarming, and require further explanation and discussion. As we have seen in our initial research on this topic, some providers who initially had a negative reaction to IUD self-removal later mentioned that they understood why it might be an important option when there were external stressors on health systems, such as COVID-19.

Finally, and most critically, more studies are needed (initially in supportive clinical settings) on what techniques work for IUD self-removal. The lone US study provided suggestions for future research, including measuring the length of strings protruding from the cervix before attempted removal (to try and gather a better idea of the length at which more self-removal attempts were successful), offering all users gloves for self-removal (and documenting whether gloves were associated with successful self-removal), and recommended positions to try (26). Documenting characteristics of successful self-removal experiences in clinical settings could subsequently inform recommendations in non-clinical settings.

Better understanding IUD self-removal presents an opportunity to integrate principles of self-care into the increasing provision of LARC, especially IUDs, in LMICs. More information is needed regarding the best techniques, success rates, and other considerations to allow IUD users to make informed decisions about self-removal and support providers in counseling and support. However, it bears repeating that self-care approaches should facilitate people's reproductive autonomy and not infringe upon it. Not every IUD user will want to self-remove, and trained providers at facilities must continue to provide removal services. Providing users with accurate information can help facilitate their ability to manage their own health, as well as enabling providers to provide support as requested, in line with the principles of self-care and reproductive autonomy.

## Author contributions

All authors contributed to the conception and design of the opinion piece. AC drafted the piece. AC, RC, MB, and LD all revised it critically for intellectual content. All authors provide approval for the publication of the content and agree to be accountable for all aspects of the piece.

## Conflict of interest

Authors AC, AM, RC, MB, and LD are employed by FHI 360.

## Publisher's note

All claims expressed in this article are solely those of the authors and do not necessarily represent those of their affiliated organizations, or those of the publisher, the editors and the reviewers. Any product that may be evaluated in this article, or claim that may be made by its manufacturer, is not guaranteed or endorsed by the publisher.
